# L-Asparaginase Activity of Fungal Endophytes from *Tabernaemontana heyneana* Wall. (Apocynaceae), Endemic to the Western Ghats (India)

**DOI:** 10.1155/2014/925131

**Published:** 2014-12-11

**Authors:** Chandramouli Manasa, Monnanda Somaiah Nalini

**Affiliations:** Department of Studies in Botany, University of Mysore, Manasagangotri, Mysore, Karnataka 570 006, India

## Abstract

“Endophytes,” the microbes residing within the plant tissues, are important sources of secondary metabolites. *Tabernaemontana heyneana* Wall., a medicinal tree, endemic to the Western Ghats with rich ethnobotanical history and unique chemical diversity, was selected to study fungal endophytes and evaluate them for L-asparaginase activity. Healthy plant parts were selected for the isolation of endophytes following standard isolation protocols. A total of 727 isolates belonging to 20 taxa were obtained. The isolates comprised of bark (11%), twig (22%), leaf (43%), fruit (12.0%), and seeds (12%). Endophytes such as *Colletotrichum, Curvularia, Fusarium, Phomopsis, Verticillium*, and *Volutella* colonized *T. heyneana* plant parts. *Fusarium* sp., *Phomopsis* spp., isolate *Thlf01*, and *Fusarium solani* were the dominant genera of bark, twig, leaf, fruits, and seed samples, respectively. The endophytes were screened for their ability to utilize L-asparagine by plate assay method. *Fusarium* spp. exhibited a high level of activity among the nine endophytes tested positive for L-asparaginase activity. Studies underline the potentials of endophyte-derived fungal L-asparaginases as sources of chemotherapeutic agents.

## 1. Introduction

Natural products derived from the medicinal plants are used across the globe as pharmaceutical drugs, cosmetics, fertilizers, insecticides, and pesticides. The overexploitation of medicinal plants is a threat to their existence with several taxa becoming extinct. Alternate sources of important metabolites have focused on the ability of microbes associated within the living tissues of plants “the endophytes” since two decades after the discovery of taxol-producing endophytic fungus* Taxomyces andreanae* from* Taxus brevifolia* [1]. All nonvascular and vascular plants examined until now have been found to harbor endophytic microbes with the potential to produce novel secondary metabolites [2]. Bioprospecting of endophytes has unraveled new molecules with therapeutic potentials. Tropics being the areas of rich biodiversity provide unique biological niches for endophytes with great diversity [3].

*Tabernaemontana* is a genus comprising of 120 species of trees and shrubs of the oleander family, Apocynaceae. Alkaloids are the predominant phytochemicals found among members of the family with 256 alkaloids characterized from* Tabernaemontana* alone [4].* Tabernaemontana heyneana* Wall. is a taxon endemic to the Western Ghats of peninsular India. The stem bark, bitter roots, flowers, and latex of fruits are used in folk medicine to treat diseases of skin and toothache and to reduce inflammation [5]. The indole alkaloids and terpenoids isolated from the roots, stem bark, fruits, and leaves possess cytotoxic, anti-implantation, and antioxidant properties [6–8].

Enzymes produced by microorganisms are employed in the treatment of cancerous cells. L-asparaginase is one such enzyme routinely employed in chemotherapy particularly for cancerous tumors of white blood cells. The enzyme deprives the cancer cells of an essential amino acid asparagine by catalyzing its breakdown ultimately leading to starvation and death. L-asparaginase is specific in its action and does not pose threat to the survival of normal cells. Bacterial asparaginases are currently in use under different trade names such as Elspar from* Escherichia coli* and Erwinase from* Erwinia chrysanthemi*. But the enzymes derived from bacteria induce mild to severe immune responses. L-asparaginase derived from eukaryotes may induce relatively least toxicity and feeble immune response [9, 10]. The production of asparaginases by fungal endophytes has been reported from plant species with anticancer potentials [11]. Since the anticancer activity has been related to the presence of the alkaloids in the plant parts of* T. heyneana*, the present study deals with the isolation of fungal endophytes and screening them for L-asparaginase activity.

## 2. Materials and Methods

### 2.1. Collection and Sampling

Bark, twig, leaf, and fruit samples (*n* = 10) were collected from a healthy tree of* T. heyneana* Wall., growing in the forests of Kodagu District, in the Talacauvery subcluster of the Western Ghats (12° 17′ to 12° 27′N and 75° 26′ to 75° 33′E), Karnataka, during the month of January, 2012. Bark samples were cut 1.5 m above ground level with a machete, swabbed in alcohol (70%, v/v). The twigs, leaves, and fruits were excised using sterile pliers. The plant samples were placed in separate polyethylene zip lock bags and stored at 4°C. The samples were transported to the laboratory and processed within 48 h of collection. A herbarium specimen of the plant is deposited in the herbarium collection of the department.

### 2.2. Surface Sterilization, Isolation, and Identification

Surface sterilization was performed by sequential immersion of samples in 70% ethanol for one min, 3.5% sodium hypochlorite (NaOCl) for three min, and rinsed three times in sterile distilled water to remove traces of sterilants [12]. The efficacy of surface sterilization was checked by pouring aliquots of the water from final rinse solutions on water agar medium (WA, 2% agar) and incubated for five days [13]. The samples were blotted dry under laminar air flow and cut into small segments of uniform size using sterile scalpel. 200 segments of bark, twig, leaf, fruit, and seeds were placed equidistantly on WA medium supplemented with streptomycin sulphate to inhibit the bacterial growth. The plates were wrapped using Clean Wrap cling film and incubated at 27°C with 12 h light and 12 h dark regimes for six to eight weeks. Colonies that emerged from tissue segments were transferred to antibiotic-free potato dextrose agar medium (PDA) to enable identification. Identification was based on morphological characters and conidial characters using standard identification manuals [14–18].

### 2.3. Data Analysis

The relative frequency of colonization (% CF) of endophytes was calculated as the number of isolates of taxon from each segment divided by the total number of segments plated × 100 [19]. Dominant endophytes expressed as percentage [20] were calculated as percentage colonization frequency divided by sum of percentage colony frequencies of all endophytes × 100. The isolation rate (IR) was calculated as the number of isolates obtained from the segments divided by the total number of segments plated and expressed as a fraction [21].

### 2.4. Screening of Endophytes for L-Asparaginase Activity

The plate assay method of Gulati et al. [22] was adopted to screen fungal endophytes for L-asparaginase activity on modified Czapek Dox's (MCD) agar medium (glucose—2.0 g/L, L-asparagine—10 g/L, potassium dihydrogen phosphate (KH_2_PO_4_)—1.52 g/L, potassium chloride (KCl)—0.52 g/L, magnesium sulphate (MgSO_4_·7H_2_O)—0.52 g/L, copper nitrate (CuNO_3_·3H_2_O)—0.001 g/L, zinc sulphate (ZnSO_4_·7H_2_O)—0.001 g/L, and ferrous sulphate (FeSO_4_·7H_2_O)—0.001 g/L) adjusted to a pH of 6.2. Phenol red indicator (0.009%) was prepared from a stock solution of 2.5% of the dye in ethanol. The control plates were prepared with MCD medium devoid of asparagine (instead containing KNO_3_—0.001 g/L as the nitrogenous source) and phenol red indicator to check the ability of test fungi to grow in the medium. The mycelial plugs from four different fungi were inoculated on MCD agar medium marked into four quadrants. The plates were incubated at 27°C for five days. The colonies exhibiting pink zones were inoculated on MCD agar medium plates to confirm the activity of enzyme prior to estimation.

### 2.5. Enzyme Estimation by Nesslerization

The positive isolates were cultured in MCD broth medium incubated at 30°C in orbital shaker (GeNei, Bangalore) set at 120 rpm for five days. L-asparaginase was estimated by Nesslerization as described by Imada et al. [23]. The reaction mixture containing 0.5 mL of 0.04 M L-asparagine, 0.5 mL of 0.5 M Tris HCl buffer (pH 8.2), 0.5 mL of enzyme, and 0.5 mL distilled water was incubated at 27°C for 30 min. 0.5 mL of 1.5 M trichloroacetic acid (TCA) was added to each reaction tube to stop the reaction. 0.1 mL was drawn from the above reaction mixture tube to another tube to which 3.7 mL of distilled water and 0.2 mL of Nessler's reagent were added and incubated for 20 min. The optical density was read at 450 nm using UV-Visible spectrophotometer (TPL Technology Pvt. Ltd., Bangalore). Blank tubes were prepared by adding the enzyme after the addition of TCA. One international unit (IU) of L-asparaginase is the amount of enzyme needed to liberate one *μ*mol/min of ammonia at 27°C [23].

## 3. Results

### 3.1. Isolation and Identification of Fungal Endophytes

**Figure 1:**
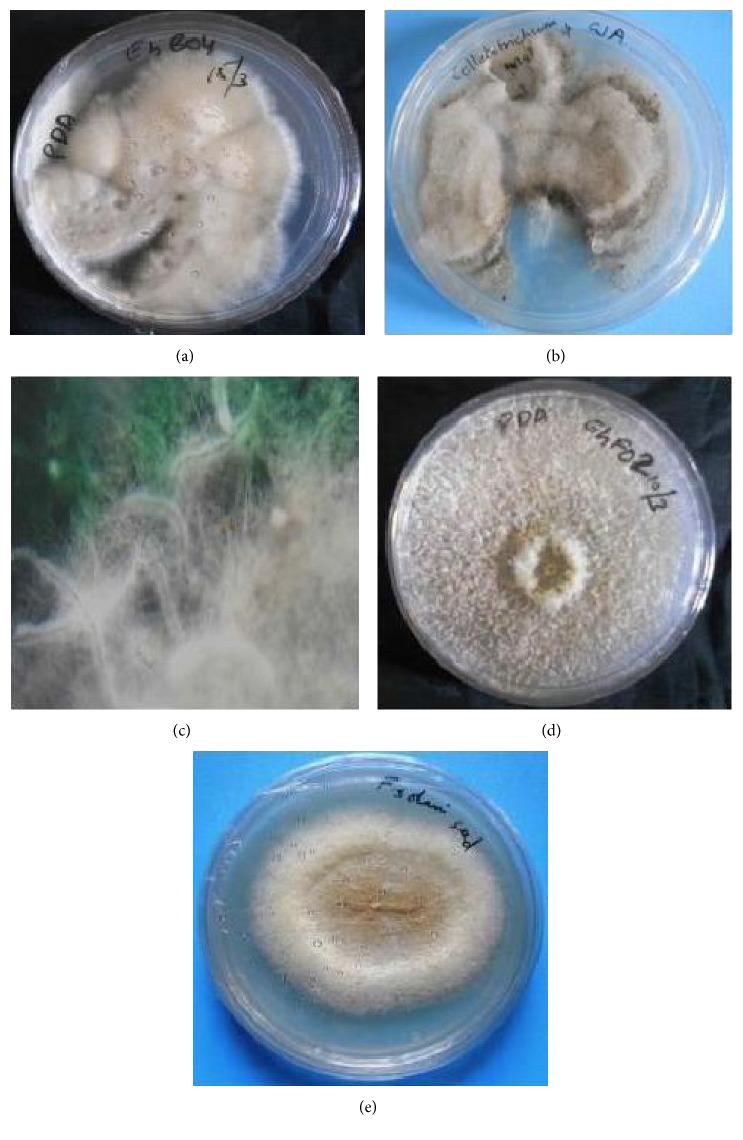
Colony morphology of dominant endophytes isolated from* T. heyneana*: (a)* F. oxysporum,* (b)* Colletotrichum* sp., (c) Isolate* Thlf01,* (d)* Phomopsis* sp., and (e)* F. solani*.

**Figure 2:**
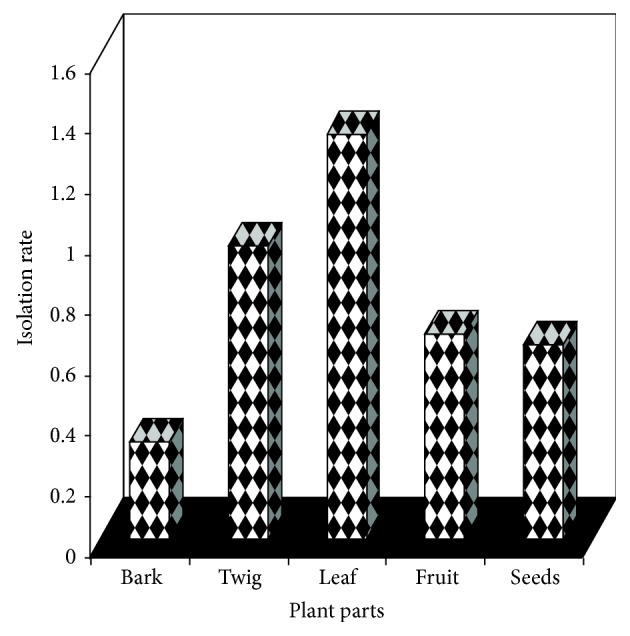
Rate of isolation of endophytic fungi from the plant parts of* T. heyneana*. Isolation rate was calculated as the number of isolates obtained from tissue segments divided by the total number of segments plated and expressed as fraction/decimal and represented.

A total of 727 isolates of endophytic fungi belonging to 20 taxa were obtained from the plating of 1000 tissue segments. The relative per cent isolation was highest for isolates of leaf samples (43%) and least for isolates of bark samples (11%). The per cent colonization frequency of endophytes differed for the plant parts used and is represented in Table 1. Many isolates belonged to the genera* Fusarium, Phomopsis,* and* Colletotrichum* which colonized more than one plant part. Tissue specificity was observed for some endophytes. Endophytes such as* Aspergillus candidus* and* Volutella* sp. were isolated from fruits only, while* Curvularia trifolii* and* Wardomyces* sp. were found to occur in seed samples and isolates of* Nectria* sp. were obtained from twig samples only. The composition and abundance of the endophytes varied for the tissues tested. The isolates of* Fusarium* were recovered from bark, twig, and seeds with three species from bark alone.* Verticillium* spp. were found to occur as isolates of bark and twig while* Colletotrichum* spp. were obtained from leaf and twig samples with more isolates being recovered from the former.* Phomopsis* spp. colonized leaf, twig, and fruit samples with 179 isolates in total. The dominant genera of bark, twig, leaf, fruit, and seeds were recorded (Figure 1).* Fusarium* spp. comprised the dominant endophytes of bark and seed samples while* Phomopsis* was the dominant genus in twig and fruit samples. The endophytic fungal isolate* Thlf01* frequently occurred in leaf samples. The isolation rate expressed in fraction for endophytes from different plant parts is represented in Figure 2. The isolation rate for leaf endophytes was more than one, thus, implying that every leaf was colonized by at least one endophytic fungus.

### 3.2. Preliminary Screening of Endophytic Fungi for L-Asparaginase Activity

**Figure 3:**
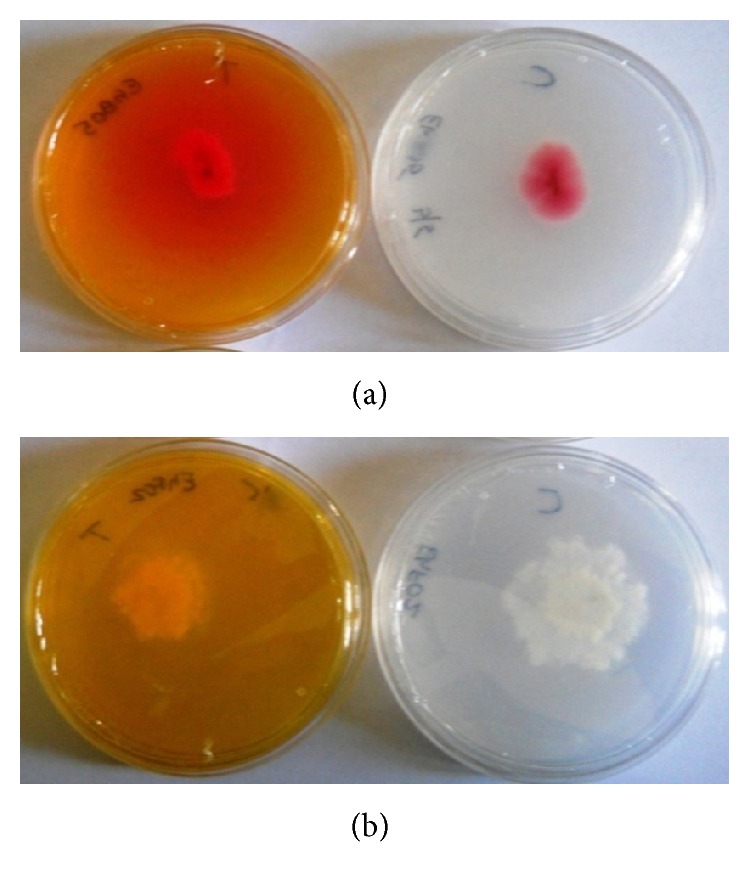
L-asparaginase activity detected by plate assay. (a) Colour change in the medium (yellow to pink) around colony indicates production of enzyme, (b) nonproducer isolate (C—control, T—test).

Preliminary screening for the enzyme activity by plate assay revealed the enzyme producing ability of nine endophytes. A pink zone was observed around the colonies suggesting that endophytic fungi were able to utilize the substrate asparagine by secreting the enzyme asparaginase which catalyzes the breakdown of the substrate (Figure 3).

### 3.3. Enzyme Estimation by Nesslerization

The reaction between the ammonia in the reaction mixture and Nessler's reagent was indicated by the formation of an orange colored solution. The enzyme activities were found to occur in the range of 0.006–1.136 unit/mL (Table 2). The isolates of* F. graminearum* from bark and twig exhibited high asparaginase activities of 0.950 IU and 0.836 IU and* F. verticillioides* showed highest activity among all the endophytic fungi with 1.136 IU of enzyme.* Verticillium* sp. and* Volutella* sp. exhibited moderate enzyme activity while* Colletotrichum* spp. exhibited least enzyme activity. The values of optical densities and enzyme activities shown by endophytes differed (Table 2).

## 4. Discussion

Research on endophytes in the past few decades has amounted to our understanding of their nature, interaction with host, and roles played by them in deterring insects, plant pathogens, and other environmental stress. Plant defense via endophytes is attributed to the production of secondary metabolites. The metabolites derived from endophytes have attracted researchers since their roles in medicine have been addressed. Tropical trees have received less attention with regard to endophytic studies in comparison to temperate trees probably due to inaccessible locations. However, in recent years several attempts have been made to bioprospect endophytes of tropical trees and have yielded fruitful outcomes. Thus, tropical regions known for the diversity of plant species also have the prospect of housing microbes with great diversity [3].

In the current investigation* T. heyneana*, a tree endemic to the Western Ghats of Southern India, was selected for bioprospecting endophytic fungi. Multiple isolates of* Colletotrichum* sp. and* Phomopsis* sp. from the twigs and leaves of* T. heyneana* were obtained.* Colletotrichum* and* Phomopsis* are reported as endophytes in previous studies from the leaves of* T. divaricata* [24, 25]. These genera are multihost endophytes because they occur consistently in taxonomically diverse tropical tree species of different geographical areas [3, 26]. The reason for the frequent occurrence of* Colletotrichum* and* Phomopsis* as endophytes may be attributed to the slimy conidia they produce which are readily dispersed by water in the rain forests [25].

The per cent colonization of endophytes was high in the leaf tissues in comparison to other plant parts. Similar observations were made by Kharwar et al. [27] in* Catharanthus roseus* where leaf segments were densely colonized with endophytes when compared to the root and stem segments. Tissue specificity observed in this study complies with the results obtained in earlier studies [27–29]. Endophytes of tropical trees show a higher degree of tissue preference than host preference. Different tissues of trees rather than the same tissue from different tree species have higher diversity of endophytes [3].

So far, 12 species of the family Apocynaceae,* namely*,* Allamanda cathartica*,* Alstonia scholaris*,* Alyxia sinensis*,* Catharanthus roseus*,* Cerbera manghas*,* Melodinus suaveolens*,* Nerium oleander*,* Plumeria acutifolia*,* Strophanthus divaricatus*,* Tabernaemontana divaricata*,* Thevetia peruviana,* and* Trachelospermum jasminoides,* have been evaluated for fungal endophytes from foliar segments, phloem (*C. roseus*), and roots [24]. The fungal endophytes of bark have been isolated from few woody plant species from the tropics such as* Azadirachta indica* [30, 31],* Crataeva magna* [32],* Terminalia arjuna* [33],* Aegle marmelos* [34], and* Prosopis cineraria* [35]. In the present study, bark, twigs, leaves, fruits, and seeds of* T*.* heyneana* were used for the study of endophytic colonization and differences among the isolates in plant parts suggested the occurrence of endophyte taxa in a single tree species.

Endophytes of* T. heyneana* examined for enzyme activity demonstrated their ability to metabolize the substrate, L-asparagine. Few reports on studies of fungal endophytes for L-asparaginase activity are available [11, 36].* Fusarium* spp. exhibited enzyme activity in the present study.* Fusarium* spp. isolated from soil and marine algae have been reported to produce asparaginase in earlier studies [10, 37].* Volutella* sp. and* V. lecanii* as endophytes with asparaginase activity has been reported in this study for the first time. Though isolates of* Fusarium* and* Colletotrichum* showed pink zones in the agar plate assay, their enzyme activities were found to be low, as estimated by the spectrophotometric method. The reason for the absence of enzyme activity in the quantitative estimation may be attributed to the differences in ability of fungi to produce enzyme in solid and liquid states [38].

L-asparaginase is produced by plants, animals, and microorganisms. The microbes are better sources of L-asparaginase because of the ease with which they can be cultured, extracted, and purified, also facilitating the industrial scale production.* E. carotovora* and* E. coli* are currently used as commercial sources of L-asparaginase. Their administration induces immune responses ranging from mild to severe in patients suffering from acute lymphoblastic leukemia. The fungal L-asparaginase has less adverse effects than bacterial L-asparaginases [39].

## 5. Conclusion

*T. heyneana*, the endemic tree species of the Western Ghats, has potential medicinal benefits. The fungal endophytes isolated from the plant parts belong to diverse taxa. Endophytes of* T. heyneana* such as* F. graminearum* have been established as producers of L-asparaginase enzyme. Further investigations involving the isolation and purification of enzyme followed by* in vitro* tests using cancer cell lines can throw light on the usefulness of the endophyte-derived L-asparaginases.

## Figures and Tables

**Table 1:** Colonization frequency of endophytic fungi from the plant parts of *T. heyneana*.

Endophytic fungi	Bark^*^	Twig^*^	Leaf^*^	Fruit^*^	Seed^*^	Total	DE (%)
*Aspergillus candidus *	—	—	—	4.5	—	9	14.07
*Curvularia trifolii *	—	—	—	—	18.5	37	28.90
*Exophiala* sp.	—	—	—	—	5.0	10	7.81
*Fusarium oxysporum *	19.5	—	—	—	—	39	61.90
*Fusarium verticillioides *	—	15.0	—	—	—	30	15.54
*Fusarium graminearum *	1.5	4.5	—	—	—	12	9.42
*Fusarium* sp.	1.5	—	—	—	—	3	4.76
*Fusarium solani *	—	—	—	—	39.5	79	61.71
*Verticillium* sp.	1.0	—	—	—	—	2	3.17
*Verticillium lecanii *	—	3.5	—	—	—	7	3.62
*Verticillium verticillioides *	—	5.0	—	—	—	10	5.18
*Volutella* sp.	—	—	—	4.5	—	9	33.33
*Wardomyces* sp.	—	—	—	—	1.0	2	1.56
*Colletotrichum dematium *	—	6.5	—	—	—	13	6.73
*Colletotrichum gloeosporioides *	—	—	44.5	—	—	89	33.20
*Colletotrichum* sp.	—	—	23.5	—	—	47	17.53
*Phomopsis vexans *	—	—	14.0	—	—	28	10.44
*Phomopsis* sp.	—	46.0	—	—	—	92	47.66
*Thlf01* ^†^	—	—	52.0	—	—	104	38.80
*Nectria* sp.	—	2.5	—	—	—	5	2.59
Number of isolates	68	133	268	80	78	627	—

Note: ^*^200 segments of each sample (*n* = 10) were plated for frequency analysis. Not detected: **—**; DE: dominant endophytes.

^†^Reference number assigned in the laboratory.

**Table 2:** Estimation of L-asparaginase production by the endophytic fungi.

Endophytic fungi	Absorbance at 450 nm	Enzyme in unit/mL
*Fusarium* graminearum^t^	0.755	1.006
*Fusarium* graminearum^b^	0.713	0.950
*Fusarium *sp.^b^	0.201	0.268
*Fusarium* *verticillioides* ^t^	0.852	1.136
*Fusarium* *oxysporum* ^b^	0.171	0.228
*Verticillium* *lecanii* ^t^	0.589	0.784
*Volutella *sp.^f^	0.478	0.637
*Colletotrichum *sp.^l^	0.048	0.064

Note: ^t^twig isolate; ^b^bark isolate; ^f^fruit isolate; ^l^leaf isolate.
